# Schaalman Senior Voices: Transitioning From “What’s the Matter?” to “What Matters?”

**DOI:** 10.1093/geroni/igaa057.1939

**Published:** 2020-12-16

**Authors:** Grisel Rodriguez-Morales, Erin Emery-Tiburcio, Robyn Golden, Marc Fenton, Jasmine Chandy

**Affiliations:** 1 Rush University Medical Center, Chicago, Illinois, United States; 2 Rush University Medical Center, CHICAGO, Illinois, United States

## Abstract

The 4Ms of an Age-Friendly Health System start with What Matters to the older adult. A unique method for asking that question is through film. Schaalman Senior Voices (SSV) films older adults talking about What Matters to them, and uses the films to stimulate discussion about later life with older adults in the community (n=264), with health care professions students learning to listen to older adults (n=1250), and health system executives considering implementation of the Age-Friendly Health Systems (AFHS) initiative (n=100). SSV has completed longer professional films interviewing 12 older adults. Using a mobile platform, SSV has filmed 50 older adults in the community and at health events. Outcomes of film discussions will be presented, including inspiration for older adults having conversations with family and physicians about What Matters, health care students effectively using skills in asking What Matters to enhance the care they provide, and executives considering AFHS implementation.

